# Guillain-Barré Syndrome As the Initial Manifestation of Human Immunodeficiency Virus Infection: A Case Report

**DOI:** 10.7759/cureus.115882

**Published:** 2026-09-07

**Authors:** Ana Albuquerque, Odete Gomes, Miguel Santos, Ana Claudia Cunha, Marta Magno

**Affiliations:** 1 Intensive Care Unit, Centro Hospitalar de Leiria, Leiria, PRT; 2 Critical Care, Unidade Local de Saúde Região de Leiria, Leiria, PRT; 3 Intensive Care Unit, Unidade Local de Saúde da Região de Leiria, Leiria, PRT; 4 Intensive Care Unit, Hospital de Santo André, Leiria, PRT

**Keywords:** acute inflammatory demyelinating polyradiculoneuropathy, autonomic dysfunction, guillain-barré syndrome, hiv, respiratory failure, syphilis

## Abstract

Guillain-Barré syndrome (GBS) is an acute immune-mediated polyradiculoneuropathy typically triggered by an antecedent infection. Human immunodeficiency virus (HIV) is an uncommon but recognized association, and GBS may rarely constitute the first clinical manifestation of previously undiagnosed HIV infection.

A 36-year-old man presented with subacute facial diplegia, predominantly distal tetraparesis, sensory ataxia, glove-and-stocking paresthesia, and hyporeflexia. Cerebrospinal fluid (CSF) analysis showed albuminocytologic dissociation; electrophysiological studies demonstrated a sensorimotor polyradiculoneuropathy with prominent demyelinating features, supporting the diagnosis of GBS. He received a five-day course of intravenous immunoglobulin (IVIG) with initial modest improvement followed by subsequent marked neurological deterioration and respiratory failure requiring invasive mechanical ventilation and a second IVIG course. Etiological investigation revealed previously undiagnosed HIV-1 infection and concomitant syphilis. Antiretroviral therapy was initiated, and presumptive neurosyphilis was treated with ceftriaxone. The patient developed autonomic instability and required tracheostomy for prolonged ventilatory weaning. He was transferred after an 89-day hospital stay with residual tetraparesis, predominantly affecting the lower limbs.

GBS may be the initial presentation leading to the diagnosis of previously unrecognized HIV infection. Concomitant syphilis added diagnostic complexity, requiring integration of clinical, electrophysiological, CSF, microbiological, and serological findings and cautious causal interpretation. This case supports considering HIV testing in otherwise unexplained acute inflammatory polyradiculoneuropathy and highlights the potential severity of HIV-associated GBS, including autonomic and respiratory complications.

## Introduction

Guillain-Barré syndrome (GBS) is an acute immune-mediated polyradiculoneuropathy affecting the peripheral nerves and nerve roots. It typically presents with progressive, relatively symmetrical limb weakness and diminished or absent deep tendon reflexes, although sensory symptoms, cranial nerve involvement, autonomic dysfunction, and respiratory insufficiency may also occur [1,2].

GBS is generally considered a post-infectious disorder. Most patients report an antecedent illness, most commonly an upper respiratory or gastrointestinal infection, before the onset of neurological symptoms. Several infectious agents have been associated with GBS, including *Campylobacter jejuni*, cytomegalovirus, Epstein-Barr virus, *Mycoplasma pneumoniae*, and human immunodeficiency virus (HIV) [1,3].

Neurological complications can occur throughout the course of HIV infection. However, GBS is an uncommon manifestation, reported during acute or early infection, chronic infection, advanced immunosuppression, and following the initiation of antiretroviral therapy (ART) as a manifestation of immune reconstitution inflammatory syndrome [3-6]. In some patients, GBS constitutes the first recognized manifestation of previously undiagnosed HIV infection [4,5].

The pathogenesis of HIV-associated GBS is not fully understood. However, it is thought to involve immune-mediated peripheral nerve injury occurring in the setting of HIV-related immune activation and dysregulation [3].

The clinical presentation of HIV-associated GBS is generally similar to that observed in patients without HIV infection. Cerebrospinal fluid (CSF) analysis in classic GBS usually shows albuminocytologic dissociation, characterized by elevated protein concentration and a normal or minimally increased white blood cell count. Mild CSF pleocytosis may occur in patients with HIV-associated GBS and does not necessarily exclude the diagnosis. However, a substantial increase in CSF cell count should prompt investigation for alternative infectious or inflammatory causes of polyradiculopathy [2,4,7].

In patients with HIV infection, the differential diagnosis also includes other HIV-associated peripheral neuropathies and infectious polyradiculopathies, particularly in the setting of advanced immunosuppression [3,4]. The acute clinical course, pattern of weakness and reflex abnormalities, CSF findings, and electrophysiological features are therefore important in distinguishing GBS from these alternative neurological disorders [2,7].

We report a case of GBS as the initial clinical presentation leading to the diagnosis of previously unrecognized HIV-1 infection, complicated by severe respiratory and autonomic involvement requiring invasive mechanical ventilation and prolonged intensive care support. Concomitant syphilis identified during the etiological investigation added diagnostic complexity. This case highlights the challenge of distinguishing an immune-mediated polyradiculoneuropathy from potentially overlapping infectious neurological manifestations when HIV and syphilis are diagnosed concurrently and emphasizes the need to integrate clinical, electrophysiological, CSF, and microbiological findings when assessing causality.

## Case presentation

A previously independent 36-year-old man with a 10-year history of depressive disorder, treated with quetiapine, escitalopram, lorazepam, and clonazepam, presented to the emergency department and was admitted to the Neurology Department on hospital day (HD) 1 with a subacute, relatively symmetrical neurological syndrome characterized by facial diplegia, predominantly distal tetraparesis, sensory ataxia, and glove-and-stocking paresthesia, with hyporeflexia in all four limbs. He was an active smoker and denied illicit drug use. Brain computed tomography (CT), performed on HD1, showed no acute intracranial abnormalities (Figure 1).

**Figure 1:**
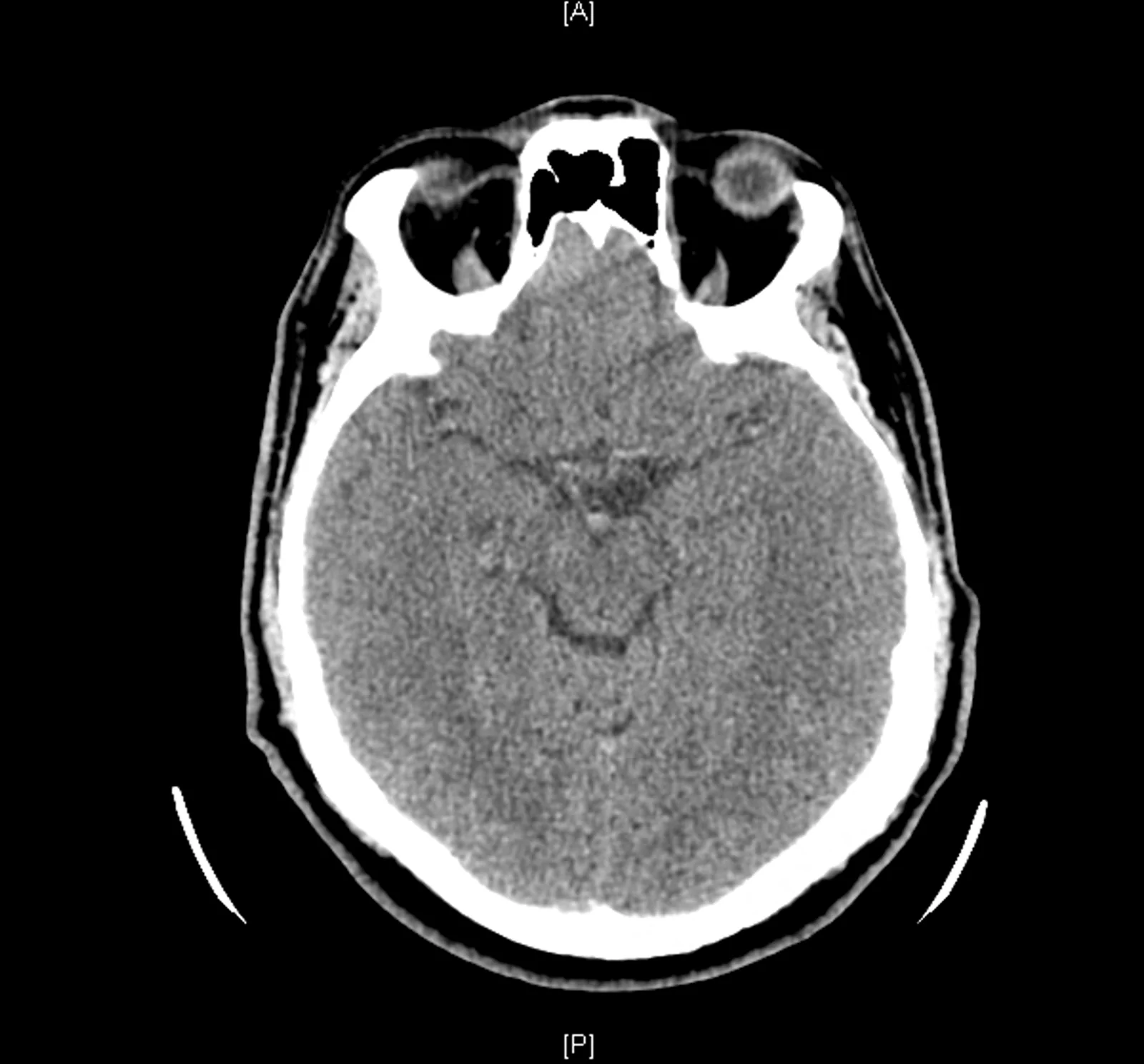
Axial non-contrast brain CT showing no acute intracranial abnormalities CT: computed tomography

Thoracolumbar spine CT, also performed on HD1, showed only mild degenerative disc and vertebral changes, without significant spinal cord or nerve root compression (Figure 2).

**Figure 2:**
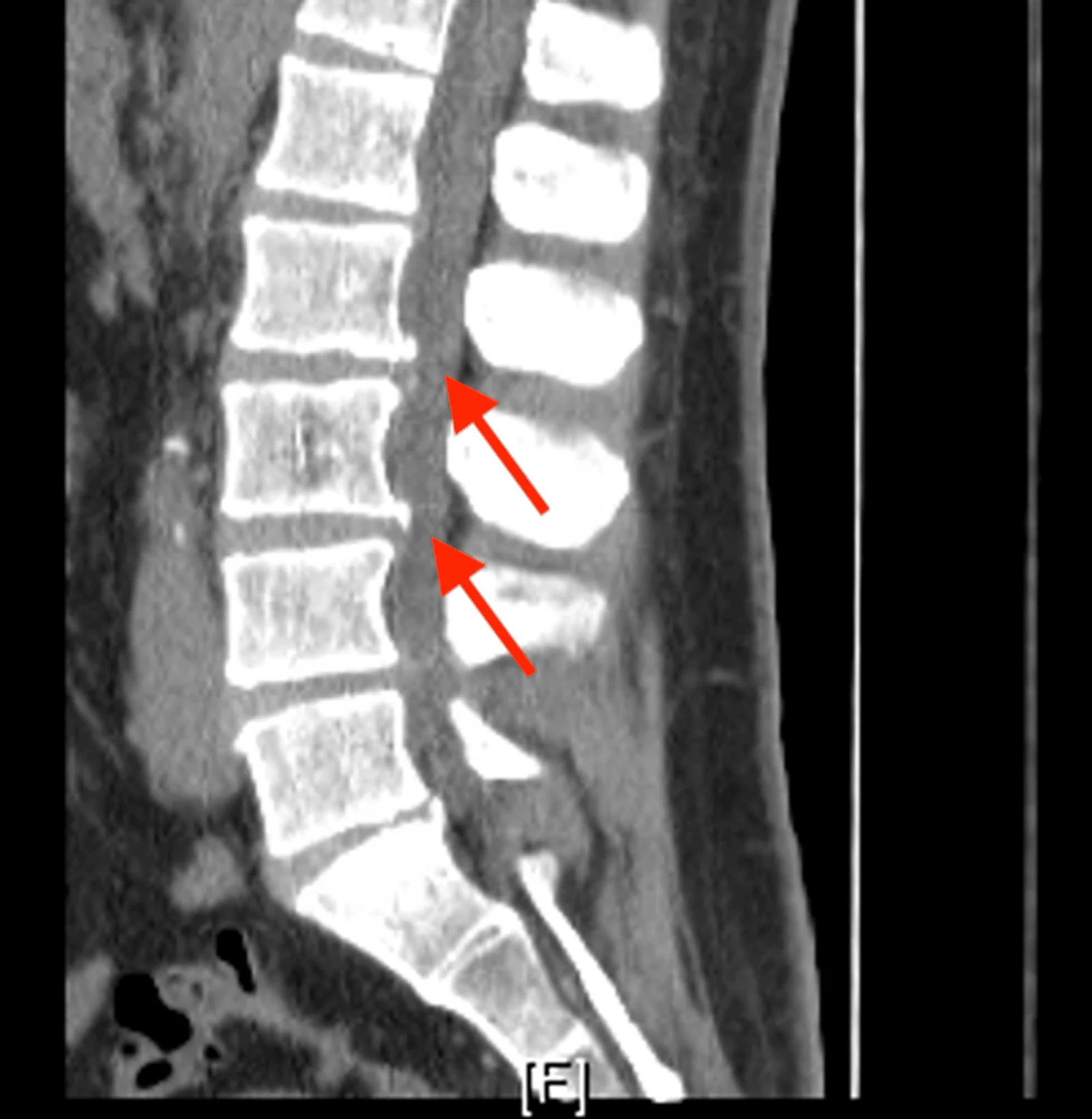
Sagittal thoracolumbar spine CT image showing mild degenerative disc changes at L3-L4 and L4-L5 (red arrows), with diffuse disc protrusions causing mild indentation of the ventral aspect of the thecal sac, without evidence of nerve root compression CT: computed tomography

CSF analysis on HD1 showed five leukocytes/mm³ (100% mononuclear), 80 erythrocytes/mm³, and a markedly elevated protein concentration of 168.9 mg/dL, with a normal glucose concentration (Table 1). Testing for bacterial capsular antigens was negative.

**Table 1:** Laboratory and CSF findings † Absolute polymorphonuclear and mononuclear cell counts were calculated from the total leukocyte count and differential reported by the laboratory (0% polymorphonuclear and 100% mononuclear cells in both samples). ALT: alanine aminotransferase, AST: aspartate aminotransferase, CSF: cerebrospinal fluid

Parameter	Initial assessment (HD1)	Repeat assessment (HD26)	Reference range
Blood			
Hemoglobin (g/dL)	16.6	11.3	13.0-17.7
Leukocytes (×10³/µL)	9.8	6.3	4.0-10.0
Lymphocytes (×10³/µL)	1.5	0.5	1.5-6.5
Platelets (×10³/µL)	219	236	150-500
Creatinine (mg/dL)	0.71	0.44	0.67-1.18
Sodium (mmol/L)	135	133	136-146
Potassium (mmol/L)	4.1	4.1	3.5-5.1
ALT (U/L)	20	32	3-45
AST (U/L)	19	18	15-50
Creatine kinase (U/L)	114	14	10-171
C-reactive protein (mg/L)	1.5	17	≤5.0
Cerebrospinal fluid			
Appearance	Clear	Clear	Clear
Leukocytes (/mm³)	5	25	0-5
Polymorphonuclear cells (/mm³)†	0	0	<5
Mononuclear cells (/mm³)†	5	25	<5
Erythrocytes (/mm³)	80	0	0
Glucose (mg/dL)	67	79	40-70
Total protein (mg/dL)	169	405	15-45
Albumin (mg/dL)	-	149	10-30

Overall, the electrophysiological findings demonstrated a sensorimotor polyradiculoneuropathy with prominent demyelinating features, including markedly prolonged distal motor latencies and reduced motor conduction velocities (Table 2, Figure 3). Given the markedly reduced compound muscle action potential (CMAP) amplitudes, the absence of sensory responses, and the lack of serial electrodiagnostic studies, a definitive electrophysiological subtype classification could not be established.

**Table 2:** Nerve conduction study findings Reference limits are those reported by the performing neurophysiology laboratory. CMAP: compound muscle action potential, NR: no response

Nerve/study	Parameter	Patient result	Reference limit
Right median motor	Distal latency	28.4 ms	≤4.6 ms
	Distal CMAP amplitude	1.0 mV	≥5.9 mV
	Conduction velocity	37 m/s	≥49 m/s
Right median sensory	Wrist-index finger response	No latency/amplitude value reported	Latency: onset ≤3.3 ms; peak ≤4.0 ms; amplitude ≥11–19 µV
Right ulnar motor	Distal latency	5.9 ms	≤3.7 ms
	Distal CMAP amplitude	2.9 mV	≥7.9 mV
	Conduction velocity	44 m/s	≥52 m/s
Right peroneal (fibular) motor	Distal latency	16.4 ms	≤6.5 ms
	Distal CMAP amplitude	0.4 mV	≥2.6 mV
	Conduction velocity	33 m/s	≥38 m/s
Right sural sensory	Response	NR	Amplitude ≥4 µV; onset latency ≤3.6 ms; peak latency ≤4.5 ms

**Figure 3:**
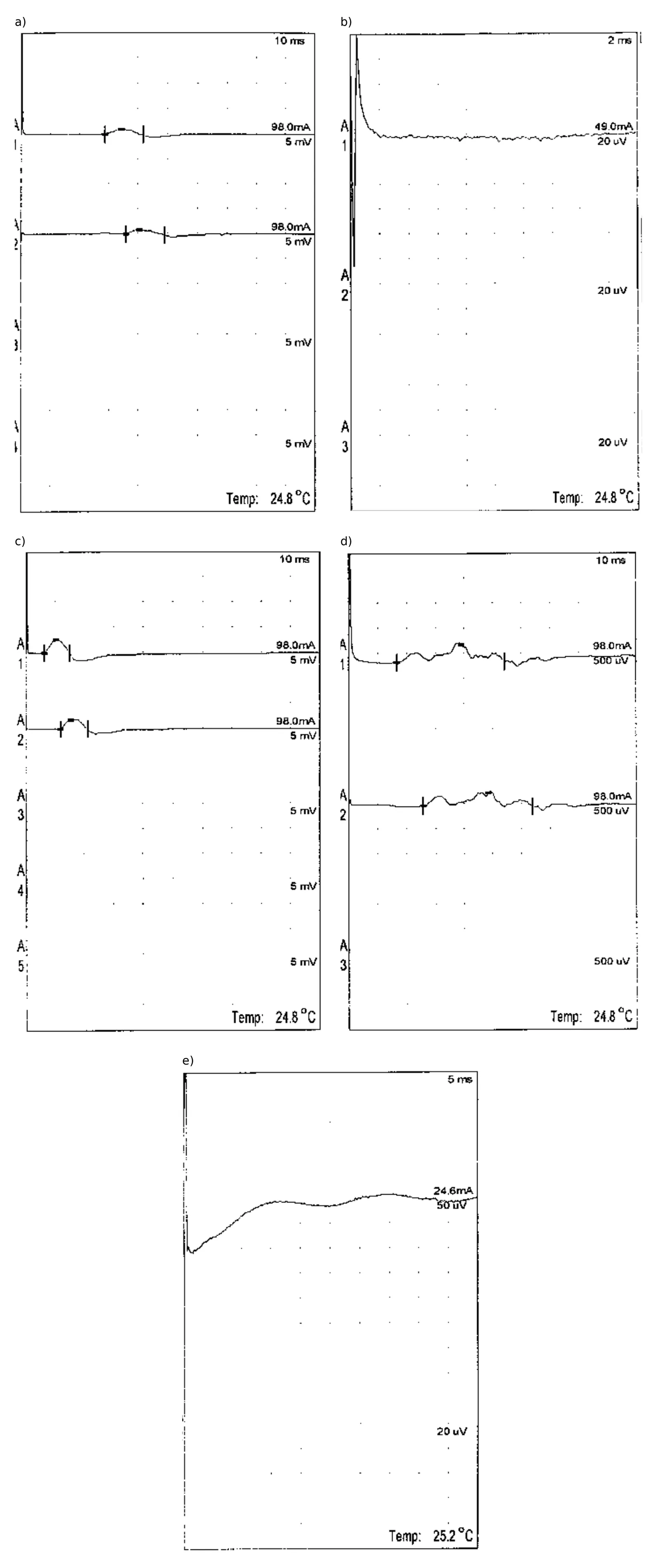
Nerve conduction study waveforms (a) Right median motor nerve conduction study showing markedly prolonged distal latency (28.4 ms), reduced CMAP amplitude (1.0 mV), and reduced conduction velocity (37 m/s). (b) Right median sensory nerve conduction study with no measurable sensory response. (c) Right ulnar motor nerve conduction study showing prolonged distal latency (5.9 ms), reduced CMAP amplitude (2.9 mV), and reduced conduction velocity (44 m/s). (d) Right peroneal motor nerve conduction study showing markedly prolonged distal latency (16.4 ms), markedly reduced CMAP amplitude (0.4 mV), and reduced conduction velocity (33 m/s). (e) Right sural sensory nerve conduction study showing no recordable sensory response. Overall, the electrophysiological findings demonstrated a sensorimotor polyradiculoneuropathy with prominent demyelinating features, supporting the clinical diagnosis of GBS. CMAP: compound muscle action potential, GBS: Guillain-Barré syndrome

The patient received a five-day course of intravenous immunoglobulin (IVIG), completed on HD5, with initially modest improvement followed by clinical stabilization. He subsequently developed marked neurological deterioration with respiratory muscle weakness, progressing to type 2 respiratory failure requiring endotracheal intubation, invasive mechanical ventilation, and admission to the intensive care unit (ICU) on HD18. After multidisciplinary reassessment with the neurology team, a second five-day course of IVIG was initiated on HD26 and completed on HD31.

During the etiological investigation, previously undiagnosed HIV-1 infection was identified. HIV-1 infection was first detected by a reactive fourth-generation antigen/antibody combination immunoassay on HD17 and subsequently confirmed by a positive INNO-LIA line immunoassay discriminating HIV-1 from HIV-2 reactivity. On HD21, the plasma HIV-1 ribonucleic acid (RNA) level was 15,404 copies/mL, and the CD4+ T-cell count was 336 cells/mm³, representing 44% of total T lymphocytes (Table 3). According to the 2014 Centers for Disease Control and Prevention (CDC) surveillance case definition for HIV infection, the patient was classified as stage 2 based on a CD4+ T-cell count of 336 cells/mm³ [8].

**Table 3:** Serological, immunological, and microbiological findings and their interpretation ANA: antinuclear antibodies, ANCA: antineutrophil cytoplasmic antibodies, CD4: cluster of differentiation 4, CMIA: chemiluminescent microparticle immunoassay, CMV: cytomegalovirus, CSF: cerebrospinal fluid, DNA: deoxyribonucleic acid, EBNA: Epstein-Barr nuclear antigen, EBV: Epstein-Barr virus, FTA-ABS: fluorescent treponemal antibody absorption, GBS: Guillain-Barré syndrome, HBc: hepatitis B core, HBs: hepatitis B surface, HBsAg: hepatitis B surface antigen, HBV: hepatitis B virus, HCV: hepatitis C virus, HIV: human immunodeficiency virus, HSV: herpes simplex virus, IgG: immunoglobulin G, IgM: immunoglobulin M, JC: John Cunningham, PCR: polymerase chain reaction, RNA: ribonucleic acid, RPR: rapid plasma reagin, s/CO: signal-to-cutoff ratio, TPHA: *Treponema pallidum* hemagglutination assay, VCA: viral capsid antigen, VDRL: Venereal Disease Research Laboratory

Test	Result	Interpretation
HIV-1 infection		
HIV-1/2 antibodies + p24 antigen, fourth-generation CMIA	Positive, 610.00 s/CO	Reactive HIV screening assay
HIV-1/HIV-2 confirmatory INNO-LIA	Positive for HIV-1	HIV-1 infection confirmed
HIV-1 RNA	15,404 copies/mL	Detectable HIV-1 viremia
CD4+ T-cell count	336 cells/mm³ (44%)	CD4 count in the 200–499 cells/mm³ category
Syphilis		
Treponemal IgG/IgM screening assay	Positive, index 35.7	Reactive treponemal screening test
RPR	Positive, titre 1:8	Reactive non-treponemal test
TPHA	Positive, titre 1:80	Reactive treponemal test
FTA-ABS total antibodies	Positive	Treponemal antibodies detected
FTA-ABS IgM	Negative	No detectable treponemal IgM
*Treponema pallidum* IgG immunoblot	Positive	Treponemal IgG antibodies detected
*Treponema pallidum* IgM immunoblot	Negative	No detectable treponemal IgM
CSF VDRL/RPR	Negative	Non-reactive CSF non-treponemal test
CSF FTA-ABS total antibodies	Positive	Treponemal antibodies detected in CSF
CSF FTA-ABS IgM	Negative	No detectable treponemal IgM in CSF
Other infectious agents associated with GBS		
*Campylobacter jejuni* IgG	13 U/mL	Negative
*Campylobacter jejuni* IgM	16 U/mL	Negative
EBV VCA IgG	>750 U/mL	Positive
EBV EBNA IgG	>600 U/mL	Positive
EBV VCA IgM	17.8 U/mL	Negative; pattern consistent with past EBV infection
CMV IgG	155 U/mL	Positive
CMV IgM	8.4 U/mL	Negative; pattern consistent with past CMV infection
HSV-1/2 IgG	>30.0 index	Positive
HSV-1/2 IgM	<0.5 index	Negative; pattern consistent with past exposure
*Chlamydophila pneumoniae* IgG	16.4 UNT	Positive
*Chlamydophila pneumoniae* IgM	4.2 UNT	Negative; no serological evidence of acute infection
*Mycoplasma pneumoniae* IgG	41.7 U/mL	Positive
*Mycoplasma pneumoniae* IgM	4.8 U/mL	Negative; pattern consistent with past exposure without evidence of acute infection
Hepatitis screening		
HBsAg	Negative, 0.29 s/CO	No serological evidence of active HBV infection
Anti-HBs	229.38 mIU/mL	Protective hepatitis B surface antibody detected
Total anti-HBc	Negative, 0.63 s/CO	No evidence of previous natural HBV infection
Anti-HCV	Negative, 0.16 s/CO	No serological evidence of HCV infection
CSF immunological and microbiological investigation		
Bacterial capsular antigens	Negative	No bacterial capsular antigens detected
Oligoclonal bands	Positive; mirrored CSF/serum pattern	Blood-brain barrier impairment without evidence of intrathecal IgG synthesis
Intrathecal IgG fraction (Reiber)	<10%	Within reference range (0–10%); no evidence of intrathecal IgG synthesis
*Borrelia* spp. DNA	Negative	No *Borrelia* DNA detected in CSF
*Herpesviridae* PCR	Negative	No herpesvirus DNA detected in CSF
HIV-1 proviral DNA	Negative	No HIV-1 proviral DNA detected in CSF
JC virus DNA	<10 copies/mL	Below assay threshold
Autoimmune screening		
ANA	Negative	Negative
ANCA	Negative	Negative

ART with raltegravir, emtricitabine, and tenofovir was initiated on HD37 following discussion with an Infectious Diseases team. By HD68, the HIV-1 RNA level had decreased to below 40 copies/mL.

The investigation also disclosed previously undiagnosed syphilis (Table 3). Serum testing showed a reactive chemiluminescent treponemal screening assay, a reactive rapid plasma reagin (RPR) test at a titer of 1:8, a positive *Treponema pallidum* haemagglutination assay (TPHA) at a titer of 1:80, positive fluorescent treponemal antibody absorption (FTA-ABS) total antibodies with negative IgM, and a positive confirmatory *Treponema pallidum* IgG immunoblot with negative IgM.

Repeat CSF analysis on HD26 showed 25 leukocytes/mm³ (100% mononuclear), a protein concentration of 405 mg/dL, and a non-reactive Venereal Disease Research Laboratory (VDRL) test, whereas the CSF FTA-ABS test was positive. Oligoclonal band analysis showed a mirrored pattern in CSF and serum with an intrathecal IgG fraction below 10%, consistent with blood-brain barrier impairment without intrathecal IgG synthesis. CSF testing for *Borrelia* species, *Herpesviridae*, HIV, and John Cunningham (JC) virus was negative (Table 3).

Given the neurological presentation, reactive serum syphilis tests, elevated CSF protein, and positive CSF FTA-ABS, presumptive neurosyphilis was considered, and the patient received a 14-day course of ceftriaxone from HD29 to HD42.

Serological testing for other pathogens classically associated with GBS, including *Campylobacter jejuni*, cytomegalovirus, Epstein-Barr virus, herpes simplex virus, *Mycoplasma pneumoniae*, and *Chlamydophila pneumoniae*, showed IgG patterns consistent with past exposure without IgM or other serological evidence of acute infection (Table 3). Antinuclear and antineutrophil cytoplasmic antibodies were negative. Hepatitis screening showed a serological pattern consistent with hepatitis B vaccination and negative hepatitis C serology.

Brain magnetic resonance imaging (MRI), performed on HD33, showed no intracranial mass, extra-axial collection, hydrocephalus, midline shift, or imaging evidence of infectious vasculitis; the major intracranial vessels were patent (Figure 4).

**Figure 4:**
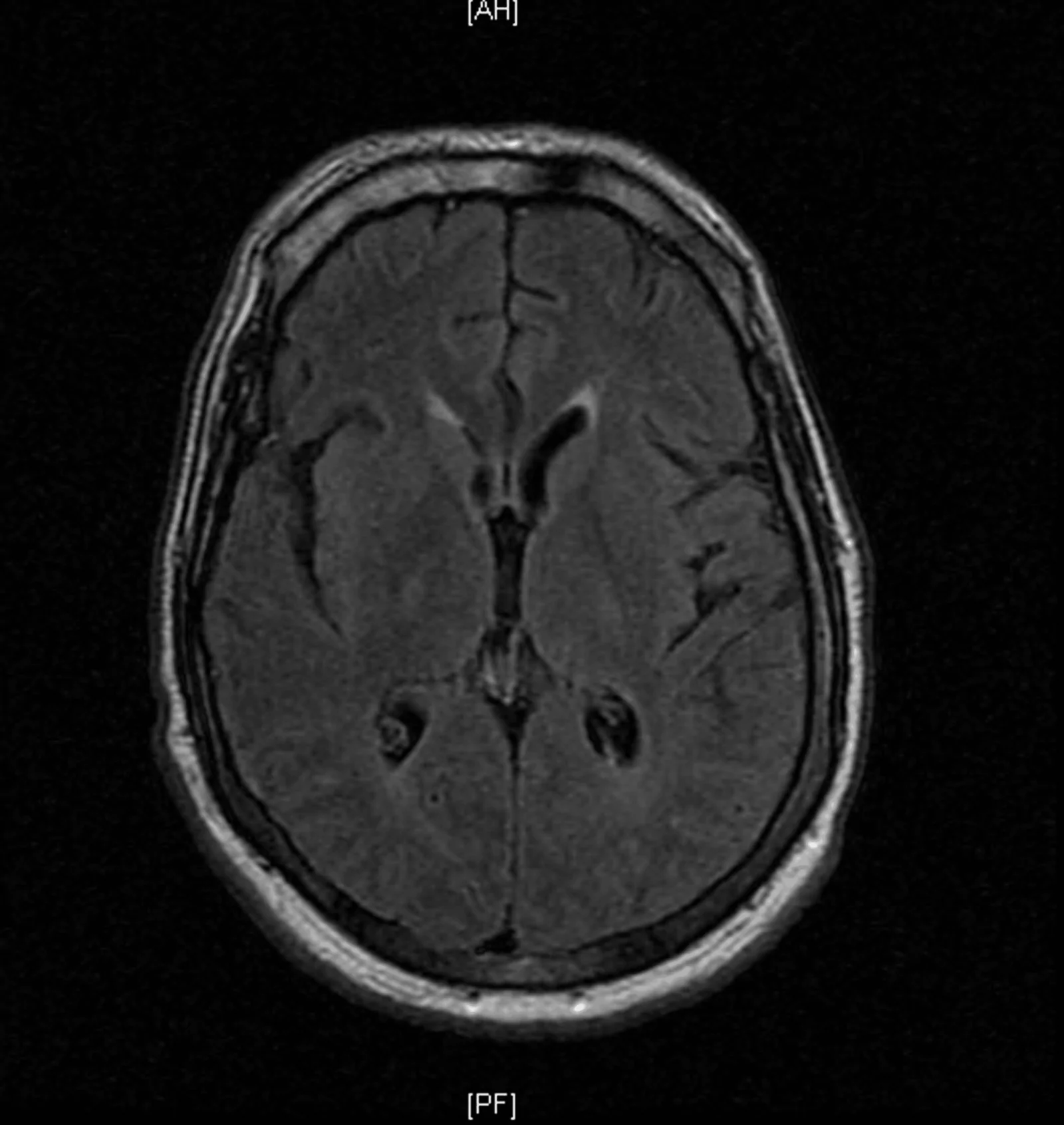
Axial T2-FLAIR brain MRI showing no significant intracranial abnormalities, with no evidence of a central nervous system lesion accounting for the neurological presentation T2-FLAIR: T2-weighted fluid-attenuated inversion recovery, MRI: magnetic resonance imaging

During the ICU stay, the patient exhibited marked hemodynamic lability, with early hypotensive episodes requiring vasopressor support followed by hypertension requiring beta-blocker therapy after sedation weaning. Transthoracic echocardiography showed no relevant structural cardiac abnormality and preserved left ventricular systolic function; sinus tachycardia was also present. This pattern was considered suggestive of autonomic dysfunction.

The ICU course was prolonged by persistent tetraparesis and diaphragmatic weakness, resulting in difficulty weaning from invasive mechanical ventilation and several episodes of ventilator-associated respiratory infection managed with targeted antibiotic therapy. A surgical tracheostomy was performed on HD44. The patient subsequently underwent progressive ventilatory weaning and commenced non-invasive ventilation on HD83.

At the time of transfer, he was awake, followed commands, and attempted to communicate. Muscle strength was grade 3/5 in the upper limbs and grade 1/5 in the lower limbs. He was breathing spontaneously with supplemental oxygen at 1 L/min during the day and receiving nocturnal average volume-assured pressure support ventilation. After an 89-day hospital stay, including 71 days in the ICU, he was transferred on HD89 to the Infectious Diseases Department of a tertiary hospital for continued treatment and neurological and respiratory rehabilitation.

## Discussion

GBS is an acute immune-mediated polyradiculoneuropathy and the most common cause of acute flaccid paralysis worldwide. Although a respiratory or gastrointestinal infection commonly precedes it, HIV is an uncommon but recognized infectious association [1,3]. GBS has been reported during acute and chronic HIV infection, advanced immunosuppression, and immune reconstitution inflammatory syndrome following the initiation of ART [3,4,6] and, in some patients, constitutes the first recognized manifestation of previously undiagnosed infection [4,5].

The present case was diagnostically challenging because GBS occurred in the setting of simultaneously diagnosed HIV-1 infection and syphilis. Both conditions may be associated with neurological manifestations [3,9], and the combination of cranial nerve involvement, reactive syphilis serology, elevated CSF protein, and a positive CSF FTA-ABS test broadened the differential diagnosis beyond an isolated immune-mediated polyradiculoneuropathy.

Nevertheless, several findings strongly supported the diagnosis of GBS. The patient presented with relatively symmetrical tetraparesis, facial diplegia, sensory symptoms, hyporeflexia, and subsequent respiratory muscle involvement. CSF analysis on admission demonstrated marked hyperproteinorrachia with only five mononuclear leukocytes/mm³, consistent with albuminocytologic dissociation.

Electrophysiological testing demonstrated markedly prolonged distal motor latencies and reduced motor conduction velocities, supporting prominent demyelinating features. However, the markedly reduced CMAP amplitudes and the absence of sensory responses, together with the absence of serial electrodiagnostic studies, precluded unequivocal classification into a specific electrophysiological GBS subtype. Current European Academy of Neurology/Peripheral Nerve Society (EAN/PNS) guidance recognizes limitations and overlap among electrophysiological GBS subtypes. It does not recommend relying on early electrodiagnostic subclassification into acute inflammatory demyelinating polyradiculoneuropathy, acute motor axonal neuropathy, or acute motor and sensory axonal neuropathy for diagnosis or treatment decisions [7].

Serological testing for other pathogens classically associated with GBS showed no evidence of acute infection, and antinuclear and antineutrophil cytoplasmic antibodies were negative. Current diagnostic approaches emphasize integrating the clinical presentation with CSF and electrodiagnostic findings rather than relying on any single diagnostic test [2,7].

Interpretation of the syphilis results requires particular caution. A reactive CSF-VDRL test is highly specific for neurosyphilis in the appropriate clinical context, but its sensitivity is limited; therefore, a non-reactive CSF-VDRL does not independently exclude neurosyphilis. Conversely, CSF FTA-ABS is more sensitive but less specific, and a positive result must be interpreted alongside the neurological manifestations, serum serology, CSF cell count and protein concentrations, and alternative diagnoses [9].

In this patient, interpretation of the positive CSF FTA-ABS required additional caution because the mirrored CSF/serum oligoclonal band pattern and intrathecal IgG fraction below 10% indicated blood-brain barrier dysfunction without evidence of intrathecal IgG synthesis. Therefore, the detection of treponemal antibodies in CSF could not, by itself, establish intrathecal antibody production or definitively confirm neurosyphilis.

In this patient, multiple concordant treponemal assays supported the diagnosis of *Treponema pallidum* infection but could not independently establish disease activity or the contribution of syphilis to the neurological syndrome. The clinical and electrophysiological pattern strongly supported GBS; however, the reactive serum syphilis tests, positive CSF FTA-ABS, neurological manifestations, and elevated CSF protein prevented neurosyphilis from being confidently excluded. Consequently, the patient received treatment for both potentially relevant processes: immunomodulatory therapy for GBS and ceftriaxone for presumptive neurosyphilis. Ceftriaxone 1-2 g daily for 10-14 days may be used as an alternative regimen for neurosyphilis, although evidence supporting alternative regimens remains limited [9].

The causal relationship between the newly identified infections and GBS cannot be established from a single case. HIV-1 may have acted as an immune trigger for the demyelinating polyradiculoneuropathy, syphilis may have contributed to the neurological presentation, or both infections may have represented concomitant conditions identified during the same etiological investigation. Accordingly, the most cautious interpretation is that GBS occurred in the setting of newly diagnosed HIV-1 infection and syphilis, with concurrent treatment for presumptive neurosyphilis, rather than unequivocally attributing the neurological syndrome to either pathogen.

Another relevant feature was the marked neurological deterioration after initial modest improvement and stabilization following the first IVIG course. This clinical evolution raised the possibility of a treatment-related fluctuation (TRF), characterized by renewed neurological deterioration after initial treatment-related improvement or stabilization [7,10,11]. Following multidisciplinary reassessment, a second IVIG course was therefore administered because the deterioration was considered a possible TRF, rather than solely based on a poor predicted prognosis. This distinction is clinically relevant because current EAN/PNS guidance recommends against routinely administering a second IVIG course solely based on poor prognosis. In contrast, retreatment with IVIG or plasma exchange may be considered in patients who experience a TRF [7]. Nevertheless, the evidence supporting retreatment in TRF remains limited, and a therapeutic effect cannot be inferred from the subsequent stabilization observed in this patient.

The patient also exhibited features suggestive of autonomic involvement, including resting sinus tachycardia and marked hemodynamic lability, with hypotensive episodes requiring vasopressor support followed by hypertension requiring beta-blocker therapy after sedation weaning. Dysautonomia is a recognized feature of severe GBS and may contribute independently to morbidity, underscoring the need for continuous hemodynamic monitoring in critically ill patients [1,2].

The prolonged ventilatory course illustrates the severity of the neuromuscular impairment. Respiratory failure required invasive mechanical ventilation from HD18, and persistent diaphragmatic weakness subsequently necessitated tracheostomy and prolonged ventilatory weaning, complicated by several episodes of ventilator-associated respiratory infection. Despite respiratory improvement, substantial motor deficits remained at transfer after a prolonged intensive care admission. Severe GBS therefore requires close respiratory and autonomic surveillance, multidisciplinary ICU management, prevention and management of complications associated with prolonged critical illness, and structured neurological and respiratory rehabilitation [2,7].

Finally, this case highlights the relevance of considering HIV testing in patients presenting with otherwise unexplained acute inflammatory polyradiculoneuropathy [3-5]. It also illustrates the diagnostic complexity posed by concomitant infections that can produce neurological manifestations. When HIV infection and syphilis are identified simultaneously, clinical, electrophysiological, microbiological, serological, CSF, and imaging findings should be integrated carefully, since no isolated finding can reliably establish the contribution of each infection to the neurological syndrome.

## Conclusions

GBS may rarely constitute the initial clinical presentation leading to the diagnosis of previously unrecognized HIV infection. In this case, the diagnosis was supported by the characteristic clinical syndrome, albuminocytologic dissociation, and electrophysiological evidence of a sensorimotor polyradiculoneuropathy with prominent demyelinating features. Concomitant syphilis complicated the etiological assessment and prompted treatment for presumptive neurosyphilis despite a non-reactive CSF-VDRL.

The subsequent development of respiratory failure, autonomic dysfunction, and prolonged ventilatory dependence illustrates the potential severity of GBS and the importance of respiratory and hemodynamic surveillance, multidisciplinary intensive care, and rehabilitation. This case also supports considering HIV testing in otherwise unexplained acute inflammatory polyradiculoneuropathy while emphasizing the necessity of cautious causal attribution when multiple potentially relevant infections are identified.
